# Inhibitory Control in Aging: A Systematic Review and Meta-Analysis of the Flanker Task

**DOI:** 10.1093/geroni/igaf122.2564

**Published:** 2025-12-31

**Authors:** Sandryne Guay, Benjamin Boller

**Affiliations:** Université du Québec à Trois-Rivières, Trois-Rivières, Quebec, Canada; Université du Québec à Trois-Rivières, Trois-Rivières, Quebec, Canada

## Abstract

The flanker task is a validated tool for measuring inhibitory control. The flanker effect is characterised by longer reaction times (RT) and lower accuracy on incongruent trials compared with congruent trials. While this effect is well established in young adults, the results for older adults remain inconsistent. The aim of this study is to carry out a systematic review and meta-analysis of the flanker effect in the elderly population. A search of PubMed, PsychInfo and PsycNet identified 22 studies comparing RTs of younger and older people using the flanker task. A meta-analysis of 3 versions of the task (arrows, cues and letters) was carried out. The results indicate that older adults systematically show slower RTs, particularly on incongruent trials, while the accuracy results remain heterogeneous. The various calculations of the cost of inhibition (subtraction, proportions and Inverse Efficiency Score) suggest that the slowing in processing speed in older adults may contribute to some extent to the differences observed. The results of the meta-analysis show a moderate effect of ageing in the version with arrows (g = 0.36), a large effect in the version with cues (g = 0.99) and a small effect in the version with letters (g = 0.22). These results highlight the impact of ageing on inhibition and suggest that the variability between studies could be explained by methodological differences. Standardizing measurement approaches will be crucial to better understand inhibitory control changes with age.

